# They Are Just Light Bulbs, Right? The Personality Antecedents of Household Energy-Saving Behavioral Intentions among Young Millennials and Gen Z

**DOI:** 10.3390/ijerph182413104

**Published:** 2021-12-12

**Authors:** Minhao Dai, Tianen Chen

**Affiliations:** 1 School of Communication and Media, Radow College of Humanities and Social Sciences, Kennesaw State University, Kennesaw, GA 30144, USA; 2 Department of Communication, Cornell University, Ithaca, NY 14850, USA; tc447@cornell.edu

**Keywords:** household energy-saving behaviors, consideration of future consequences, environmental value orientation, individualism and collectivism, regulatory focus, self-monitoring

## Abstract

Small individual behaviors such as household energy-saving behaviors may have major environmental impacts. Individuals may combat global warming by replacing traditional light bulbs with more energy-efficient light bulbs such as LED bulbs, which save electricity and reduce greenhouse gas emissions. Guided by the integrative model of behavioral prediction, the current study explored the effects of five individual personality differences (i.e., consideration of future consequences, environmental value orientation, individualism and collectivism, regulatory focus, and self-monitoring) on young Millennials’ and Gen Z’s attitudes, perceived norms, perceived control, and intention to switch light bulbs. The results of a survey indicated that environmental value orientation, individualism and collectivism, regulatory focus, and self-monitoring all significantly predicted attitudes, perceived norms, and perceived control, which predicted behavioral intention. The findings suggested the complex psychological nuance of environmental protection behaviors, even among the “greenest” generations. Implications and directions for future studies were discussed.

## 1. Introduction

Small steps can lead to big changes; this cannot be more true about energy conservation and environmental protection. During the last century, the average global surface temperature has increased approximately 2 °F (1.11 °C) [1], and it is predicted that the temperature will rise by 2–6 °C by the end of this century [2]. Most publishing climate scientists agree that global warming is caused by human-induced greenhouse gas (e.g., carbon dioxide) [3]. Greenhouse gas emissions are produced by production and consumption activities by individuals, enterprises, and institutions [4]. To reduce the destructive impacts on the environment and ease the threat of global warming, it is important for individuals to engage in environmentally friendly behaviors [5]. Individuals can alleviate the current situation of global warming by participating in conservation behaviors such as planting trees [6] or replacing conventional light bulbs with more energy-efficient light bulbs, a one-time action that has been proved to be effective at reducing carbon dioxide emissions through saving electricity [7]. Using light-emitting diode (LED) light bulbs for buildings and outdoor spaces reduced an estimated 570 million tons of carbon dioxide emissions in 2017 [8]. Many often underestimate the significant environmental impacts that small individual behaviors can make, so understanding the complex socio-psychological mechanism behind “simple” individual environmental protection behaviors can benefit public promotion efforts.

While the literature has examined the effects of a variety of social, behavioral, and psychological factors on environmental protection behaviors, it has been mostly data-driven, rather than theoretically grounded. To fill this gap in the current literature, we applied the integrative model of behavioral prediction (IMBP) to examine the effects of five personality antecedents (i.e., consideration of future consequences (CFC), environmental value orientation, individualism and collectivism, regulatory focus, and self-monitoring) on young millennials’ and Gen Z’s attitudes, perceived norms, perceived control, and behavioral intention to replace conventional light bulbs with energy-efficient light bulbs. We hoped the theoretical framework would render a more systematic and comprehensive understanding of this household energy-saving behavior. “Young millennials” and “Gen Z” refer to individuals who were born between the early 1990s and the early 2000s [9]. We focused on young millennials and Gen Z not only because they are more likely to perceive global warming as personally important and relevant [10], but also because they are the driving force of environmental activism [11]. The current study results will hopefully contribute to our understanding of what drives household energy-saving behaviors among these younger adults, shed light on the application of IMBP in environmental contexts, and inform policymakers and activists in the field of environmental protection.

## 2. Literature Review

### 2.1. Integrative Model of Behavioral Prediction

Developed based on the theory of reasoned action, the IMBP [12] is a model that can be applied to understand, explain, and predict a rational or “planned” behavior. The IMBP suggests that three types of behavior-specific perceptions determine behavioral intention: attitudes, perceived norms, and perceived control [13]. Attitudes are defined as general evaluations about the outcome of performing one given behavior, and comprise specific behavioral beliefs and outcome evaluations [13]. Perceived norms are defined as one’s general beliefs about the social pressure on performing the given action, and comprise injunctive norms and descriptive norms [14]. Perceived control is defined as general beliefs about one’s ability to perform the given behavior, and comprises specific beliefs about the enablers of and the barriers to performing the behavior and the frequency of encountering them [13,14]. Attitudes, perceived norms, and perceived control directly predicted behavioral intention [14,15]. Fishbein [12] suggested that background variables (e.g., personalities and culture) may influence individuals’ attitudes, perceived norms, and perceived control of one given behavior. The reasoned action theories (e.g., the theory of planned behaviors (TPB)) have been widely employed in ecological contexts. For instance, in a survey study, Gibson et al. [16] applied the TPB to explore the determinants of US residents’ behavioral intention to conserve water resources; the results indicated that attitudes, subjective norms, and perceived behavioral control were positively correlated with behavioral intention. Kaiser et al. [17] surveyed 468 university students and found that attitudes, subjective norms, and perceived behavioral control significantly predicted their behavioral intention to perform conservation behaviors (e.g., stop using a clothes dryer), providing support for the explanatory power of TPB. In the current study, we expanded upon the background variables in the IMBP by exploring the relationships between five personality antecedents and environmental protection behaviors, which were CFC, environmental value orientation, individualism and collectivism, regulatory focus, and self-monitoring. These five variables were separately considered in previous studies on environmental beliefs and behaviors [18,19,20,21,22,23]. However, these personality antecedents were often treated as the direct predictors of intention and behavior [24]. These analytical propositions did not align with how the IMBP viewed personality antecedents. Thus, the current study examined these personality traits as the antecedents of the three IMBP beliefs (i.e., attitudes, perceived norms, and control beliefs) and how these IMBP beliefs subsequently predicted behavioral intention.

### 2.2. Consideration of Future Consequences

CFC can be defined as “the extent to which individuals consider the potential distant outcomes of their current behaviors and the extent to which they are influenced by these potential outcomes. It involves the intrapersonal struggle between present behavior with one set of immediate outcomes and one set of future outcomes” [25]. There is a healthy number of studies that have assessed the associations between CFC and environmentally friendly attitudes, beliefs, intentions, and behaviors [26,27,28,29]. For example, Khachatryan et al. [29] conducted an online study to examine the links between CFC and fuel preference (i.e., gasoline vs. biofuels), and the study results suggested that CFC was positively related to preference for biofuels. Enzler [27] analyzed the associations between CFC and 17 self-reported environmentally friendly behaviors (e.g., switching off lights when leaving the room), and the results indicated that CFC was a valid predictor of environmentally friendly behaviors. To date, scholars have yet to assess the relationship between CFC and switching light bulbs, to the best of our knowledge.

### 2.3. Individualism and Collectivism

Cultural traits such as individualism and collectivism also have the potential to predict people’s environmentally friendly attitudes and behaviors [30]. Individualists have “a preference for a loosely-knit social structure in which individuals take care of themselves and their immediate families only” [31], whereas collectivists believe that “groups bind and mutually obligate individuals” [31]. Collectivists tend to focus on the continued existence and are more closely connected to the society and surrounding environment, so they are more likely to have positive environmental attitudes and perform environmentally friendly behaviors than individualists [30]. Many studies have examined the links between environmentally friendly behaviors and collectivism and individualism. For example, Higueras-Castillo et al. [32] surveyed people in three different countries to explore the link between collectivism and environmentally friendly behaviors, and the results indicated that collectivism was positively correlated with environmentally friendly behaviors. Kim and Choi [20] found that collectivism had indirect positive impacts on green purchase behavior via perceived consumer effectiveness. Results from a recent study [33] found that more individualism-oriented individuals were less likely to take actions to combat climate change than more collectivism-oriented ones.

Triandis and Gelfand [34] suggested that both collectivism and individualism could be either horizontal or vertical. People who score high on vertical collectivism perceive themselves as part of the group, are willing to accept the hierarchy and inequality within the group, and submit to the authorities; whereas people who score high on horizontal collectivism perceive all group members as similar and equal, and they do not submit to the authorities easily. Vertical individualists see themselves as fully autonomous; they recognize that inequality exists among individuals and accept it. Horizontal individualists also see themselves as fully autonomous, but they place emphasis on equality [34]. Cho et al. [35] found that horizontal and vertical collectivism were positively related to environmentally friendly attitudes, which influenced environmental commitment. Cho et al. [35] also hypothesized that horizontal and vertical individualism had indirect negative impacts on environmental commitment, but their study’s results did not support such hypotheses. Ali et al. [36] found that horizontal and vertical collectivism positively moderated the relationship between social status motivation and intention to purchase a green luxury car.

### 2.4. Regulatory Focus

Regulatory focus (i.e., prevention focus and promotion focus) [37] could also affect individuals’ environmentally friendly behaviors [38]. Individuals with a high prevention focus tend to focus on avoiding undesirable outcomes while achieving safety and security, whereas individuals with a high promotion focus tend to focus on achieving desirable outcomes such as accomplishments and aspirations [39]. A number of studies have examined the associations between regulatory focus and environmentally friendly behaviors, but the directions of the associations were not always consistent. Specifically, Chen et al. [38] surveyed 847 participants and found that both prevention and promotion focuses were positively related to individuals’ waste-recycling behavior. Bhatnagar and McKay-Nesbitt [40] found that promotion focus was a positive predictor of individuals’ environmental concern, while prevention focus was not. Zou and Chan [41] conducted two studies to explore the associations among regulatory focus, ethical ideology, environmentally friendly intention, and behaviors (i.e., recycling and bringing shopping bags). The results indicated an indirect positive association between prevention focus and intention and behaviors and an indirect negative association between promotion focus and the outcome variables [41].

### 2.5. Environmental Value Orientation

Previous studies on environmental protection suggested that environmental value orientation could potentially predict people’s beliefs and intention to perform environmentally-friendly actions [18,42]. A value can be defined as “a desirable trans-situational goal varying in importance, which serves as a guiding principle in the life of a person or other social entity” [43]. There are three types of value orientations related to environmental protection: ecocentrism, anthropocentrism, and environmental apathy. Ecocentrism suggests that nature should be protected because it has independent value and deserves protection for its own sake. Ecocentric individuals will take actions to protect the environment even if the actions involve discomfort, inconvenience, and expense [44]. Anthropocentrism is related to the belief that nature should be protected because of its contributions to human materialistic goals [44]. Environmental apathy refers to the carelessness or lack of motivation to protect or maintain the environment [45].

The literature has examined the associations between environmental value orientation and environmentally friendly beliefs and behaviors. For example, Casey and Scott [46] surveyed 292 individuals and found that there was a positive association between the frequency of performing environmentally friendly behaviors and ecocentrism and a negative association with environmental apathy. Kaida and Kaida [47] found that both ecocentric and anthropocentric values were positively associated with environmentally friendly behaviors. Tezel and Giritli [48] found that ecocentric values were positively correlated with environmental beliefs and awareness and that ecocentric values also predicted environmentally friendly workplace behavior. The overall research has established the positive correlation between ecocentric and anthropocentric values and environmental protection behaviors, as well as the negative correlation between environmental apathy and environmental protection behaviors.

### 2.6. Self-Monitoring

Self-monitoring is a personality construct that refers to how much individuals observe and control their self-presentation and expressive behavior [49]. An individual who scores high on self-monitoring is willing to be and is “skilled at controlling and modifying his social behavior and emotional expression to suit his surroundings on the basis of cues in the situation which indicate what attitudes and emotions are appropriate” [49]. Hartmann and Apaolaza-Ibanez [50] suggested that self-expression might be positively related to consumers’ intention to purchase green-branded electricity because performing such behavior would help them to signal their altruism and pro-social and environmentally friendly orientation. Kabadayı et al. [51] also suggested that high self-monitoring individuals had greater concerns for social and contextual appropriateness of their expressive behaviors, so they were more likely to engage in green consumption behaviors.

### 2.7. Current Study

As demonstrated above, previous studies have separately examined the links between these personality antecedents (i.e., CFC, environmental value orientation, individualism and collectivism, regulatory focus, and self-monitoring) and environmentally friendly behaviors. Many of these studies were data-driven and treated these antecedents as direct predictors of intention or behavior. To examine the associations between these antecedents and environmentally friendly intentions in a more systemic and comprehensive way, we proposed the following hypotheses and a research question based on the theoretical propositions of the IMBP and the current literature:

**Hypothesis** **1** **(H1):** *Young millennials’ and Gen Z’s attitudes, perceived norms, and perceived control towards switching to energy-efficient light bulbs would be positively associated with their behavioral intention.*

**Hypothesis** **2** **(H2):** *(a) CFC, (b) ecocentric value, (c) anthropocentric value, (d) horizontal and vertical collectivism, and € self-monitoring would positively predict young millennials’ and Gen Z’s attitudes, perceived norms, and perceived control towards switching to energy-efficient light bulbs.*

**Hypothesis** **3** **(H3):** *(a) Environmental apathy and (b) horizontal and vertical individualism would negatively predict young millennials’ and Gen Z’s attitudes, perceived norms, and perceived control towards switching to energy-efficient light bulbs.*

**Research** **Question** **1** **(RQ1)**: *How would (a) promotion focus and (b) prevention focus predict young millennials’ and Gen Z’s attitudes, perceived norms, and perceived control towards switching to energy-efficient light bulbs?*

## 3. Methods

We administrated a cross-sectional online questionnaire with young millennials and members of Gen Z. The questionnaire items assessed participants’ culture traits (i.e., individualism and collectivism), CFC, regulatory focus (i.e., prevention focus and promotion focus), environmental value orientation (i.e., ecocentrism, anthropocentrism, and environmental apathy), self-monitoring, IMBP variables (i.e., attitudes, norms, perceived behavioral control, and intention), and demographic variables (e.g., gender and ethnicity). Participants (*n* = 563) comprised young millennial and Gen Z students from an undergraduate research participant pool at a large Midwestern university. The participants were all between the age of 18 and 25, and most of the participants were female (*n* = 390, 69.3%). The majority of participants identified as White (*n* = 454, 80.6%); 46 participants (8.2%) identified as Black or African American; 29 participants (5.2%) identified as Asian; 14 participants (2.5%) identified as Latinx; and 20 participants (3.6%) identified as others. The participants received a small portion of course credits after completing the survey. Informed consent was collected from all participants, and the institutional review board approved all procedures.

### 3.1. Measures

#### 3.1.1. Consideration of Future Consequences

CFC was assessed using 12 items (e.g., “I consider how things might be in the future and try to influence those things with my day to day behavior” and “Often I engage in a particular behavior in order to achieve outcomes that may not result for many years”) [52]. Participants were asked to rate these items as extremely uncharacteristic or extremely characteristic (7-point Likert-type scale). Some items were reversed to where higher scores indicated more consideration of future consequences. The 12 items formed a reliable measure (*α* = 0.75), and the mean of the 12 items was 4.48 (*SD* = 0.72).

#### 3.1.2. Individualism and Collectivism

Sixteen items were used to assess individualism and collectivism using a 7-point Likert-type scale [34]. Four items measured horizontal individualism (e.g., “My personal identity, independent of others, is very important to me”; *α* = 0.75, *M* = 5.35, *SD* = 0.97); four items measured vertical individualism (e.g., “Winning is everything”; *α* = 0.70, *M* = 4.35, *SD* = 1.04); four items measured horizontal collectivism (e.g., “The well-being of my coworkers is important to me”; *α* = 0.77, *M* = 5.52, *SD* = 0.87); and four items measured vertical collectivism (e.g., “It is important to me that I respect the decisions made by my groups”; *α* = 0.70, *M* = 5.21, *SD* = 0.93). Higher scores on each subscale indicated stronger values on each subscale. 

#### 3.1.3. Regulatory Focus

Eleven items were used to assess regulatory focus on a 7-point Likert-type scale [53]. Six items measured promotion focus (e.g., “How often have you accomplished things that got you ‘psyched’ to work even harder?”; *α* = 0.70, *M* = 4.72, *SD* = 0.78), and five items measured prevention focus (e.g., “How often did you obey rules and regulations that were established by your parents?”; *α* = 0.79, *M* = 4.51, *SD* = 1.04). Higher scores on each subscale indicated stronger values on each subscale.

#### 3.1.4. Environmental Value Orientation

Thirty-three items were used to assess environmental value orientation on a 7-point Likert-type scale [44]. Twelve items measured ecocentric value (e.g., “Sometimes it makes me sad to see forests cleared for agriculture; *α* = 0.86, *M* = 5.52, *SD* = 0.87); twelve items measured anthropocentric value (e.g., “One of the most important reasons to keep lakes and rivers clean is so that people have a place to enjoy water sports”; *α* = 0.72, *M* = 4.26, *SD* = 0.72); and nine items measured environmental apathy (e.g., “Too much emphasis has been placed on conservation”; *α* = 0.89, *M* = 3.05, *SD* = 1.13). Items for each subscale were summed and divided. Higher scores on each subscale indicated stronger values on each subscale.

#### 3.1.5. Self-Monitoring

We used 25 items to assess self-monitoring (e.g., “Even if I am not enjoying myself, I often pretend to be having a good time”) [49]. Participants were asked to rate each item as true or false. We scored responses to each item to be either correct (scored 1) or incorrect (scored 0). The total scores were then combined, and each participant received a sum score. The possible range of scores was between 0 and 25. The average score for the 25 items was 6.53 (*SD* = 2.74).

#### 3.1.6. IMBP Variables 

These constructs included attitudes, perceived norms, and perceived behavioral control (PBC) beliefs, and intention, and were constructed based on Fishbein and Ajzen’s [13] recommendations. Attitudes were measured using eight items on a 7-point semantic differential scale (e.g., “good vs. bad”). One item was reverse-scored to where higher scores indicated more favorable attitudes towards switching light bulbs. The eight items formed a reliable measure (α = 0.88), and the mean of the eight items was 5.85 (*SD* = 1.00). Norms were measured using three items on a 7-point Likert-type scale (e.g., “Most people who are important to me think I should replace standard light bulbs with more energy-efficient options”). Higher scores indicated more favorable perceived norms towards switching light bulbs. The three items formed a reliable measure (α = 0.90), and the mean score of the three items was 4.62 (*SD* = 1.17). Perceived behavioral control was measured using three items on a 7-point Likert-type scale (e.g., “Whether or not I replace standard light bulbs with more energy-efficient options is completely up to me”). Higher scores indicated more perceived control over switching light bulbs. The three items formed a reliable measure (α = 0.79), and the mean of the three items was 5.30 (*SD* = 1.06). Intention was measured using four items on a 7-point Likert-type scale (e.g., “I am willing to replace standard light bulbs with more energy-efficient options”). Higher values indicated stronger intention. The four items formed a reliable measure (α = 0.92), and the mean of the four items was 4.98 (*SD* = 1.16). The full list of items in each variable is provided as a Supplementary File S1.

### 3.2. Analysis Plans

The sample size for the structural equation model (SEM) was determined through the ratio of observations to estimated parameters (*N*:*q*), following Schreiber et al.’s [54] guidelines. After the data were collected, we first checked the data assumptions of the dataset, including skewness, kurtosis, and multicollinearity. The steps we used to prepare the dataset for consequential analyses were presented in Section 4. We tested the relationships between observed and latent variables using exploratory factor analysis (EFA). We built a full measurement model in which all variables could freely associate with each other. This model included four latent IMBP variables: four intention items loaded onto latent intention; eight attitudinal beliefs loaded onto latent attitudes; three norms items loaded onto latent norms; and three perceived control items loaded onto latent perceived behavioral control. We further included ten background variables: twelve items loaded onto consideration of future consequences; four items loaded on horizontal individualism; four items loaded onto horizontal collectivism; four items loaded onto vertical individualism; four items loaded onto vertical collectivism; six items loaded onto promotion focus; five items loaded onto prevention focus; twelve items loaded onto ecocentric; twelve items loaded onto anthropocentric; and nine items loaded onto environmental apathy. The composite self-monitoring was entered into the model as an observed variable, as it was a dichotomous grade-based variable. We used standardized factor loadings to identify potential issues with the full measurement model. The full list of survey item abbreviations and full questions was provided as a supplementary document in submission. To test the hypotheses and answer the research question, we used the results of the final measurement model to build an SEM. We reflected the theoretical propositions of the IMBP in our SEM: all the personality antecedents predicted each of the three IMBP beliefs (i.e., attitudes, norms, perceived behavioral control), and the three beliefs predicted intention. We used maximum likelihood (ML) estimation in AMOS 26 to test the EFA and SEM. We used the guidelines offered by Schreiber et al. [54] when reporting both measurement and structural models.

## 4. Results

### 4.1. Data Preparation

We first checked the kurtosis and skewness of each endogenous variable. The skewness scores of all endogenous variables were within the range of −3 and 3; the kurtosis scores of all endogenous variables were within the range of −3 and 3. No correlations among the two variables were greater than 0.94. We then computed the variance inflation factor (VIF) to check any potential multicollinearity between exogenous variables. The VIF of exogenous variables was all below 3, which indicated that there was no significant issue with multicollinearity. Thus, we did not transform any variables in the dataset and analyzed the data.

### 4.2. Measurement Model

We computed an EFA model based on the model specifications presented in Section 3.2. The model fit indices presented an acceptable fit to the data, *χ*^2^ (3900, *n* = 563) = 8279.99, *χ*^2^/*df* = 2.12, *p* < 0.001, *CFI* = 0.88, *RMSEA* = 0.04 (90% CL = 0.04–0.04, *PCLOSE* = 0.00). Upon the examination of the standardized factor loadings, we found several items that did not meet the absolute value of the 0.40 threshold. These items were CFC5 (*β* = 0.19), CFC8 (*β* = 0.10), RF1 (*β* = 0.29), VO14 (*β* = 0.29), and VO22 (*β* = 0.16). The information related to the abbreviations can be found in the supplementary file. These items were dropped from the measurement model. The model fit of the final EFA model, *χ*^2^ (3725, *n* = 563) = 7669.26, *χ*^2^/*df =* 2.06, *p* < 0.01, *CFI* = 0.90, *RMSEA* = 0.04 (90% CL = 0.03–0.04, *PCLOSE* = 0.00), improved slightly. As there were a total of 90 items, presenting the final EFA results in a figure was not feasible; we present the factor loadings of each latent variable in Table 1 and the standardized covariances between each latent variable in Table 2.

### 4.3. Structural Model

**Figure 1:**
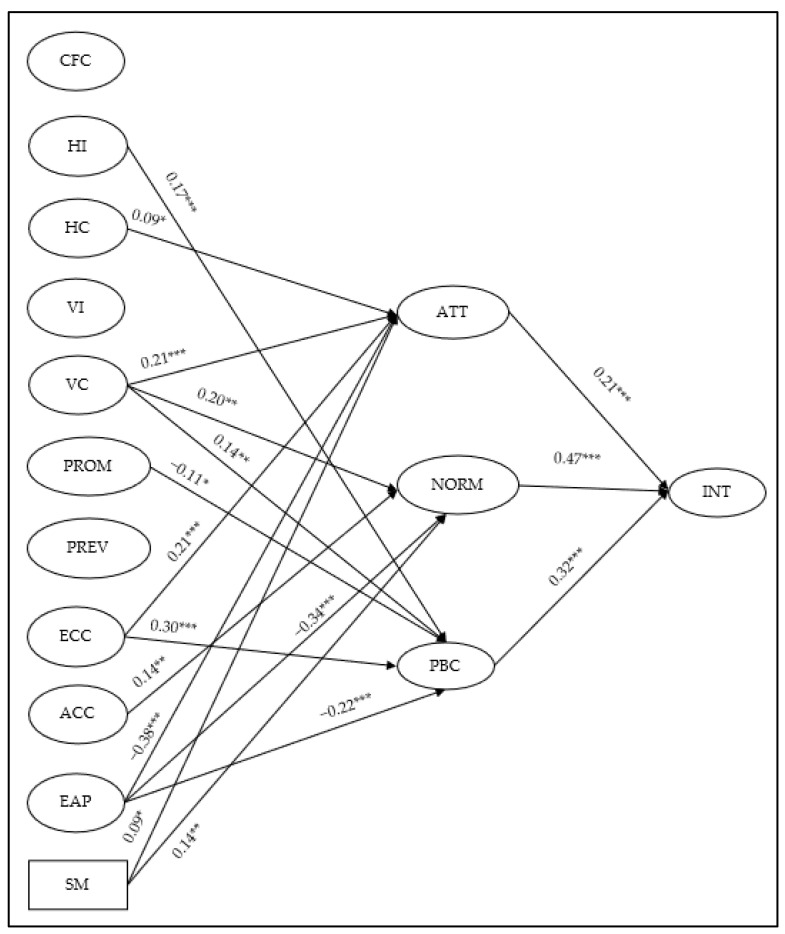
SEM with significant paths. Notes: All shown paths are statistically significant with standardized path coefficients. * indicates significant at 0.05 level; ** indicates significant at 0.01 level; *** indicates significant at 0.001 level. The path coefficients can be found in Table 3. The circles refer to latent variables; the rectangular refers to an observed variable. INT = intention, ATT = attitudes, PBC = perceived behavioral control, HI = horizontal individualism, VI = vertical individualism, HC = horizontal collectivism, VC = vertical collectivism, CFC = consideration of future consequences, PROM = promotion regulatory focus, PREV = prevention regulatory focus, ECC = ecocentric, ACC = anthrocentric, EAP = environmental apathy, SM = self-monitoring.

We specified the SEM based on Section 3.2. We used ML estimation, and allowed the estimations of means and intercepts to accommodate the missing data. The model fit indices presented an acceptable fit to the data, *χ*^2^ (3969, *N* = 563) = 9664.95, *χ*^2^/*df* = 2.44, *p* < 0.001, *CFI* = 0.85, *RMSEA* = 0.05 (90% CL = 0.04–0.05, *PCLOSE*= 0.25). We then used the standardized path coefficients and their significance values between latent variables (with observed self-monitoring) to examine the structure of the SEM. We visually present the SEM in Figure 1, and only the statistically significant paths are presented for visual clarity. The results showed that horizontal collectivism (*β* = 0.09, *p* < 0.05), vertical collectivism (*β* = 0.21, *p* < 0.001), ecocentric (*β* = 0.21, *p* < 0.001), environmental apathy (β = −38, *p* < 0.001), and self-monitoring (β = 0.09, *p* < 0.05) all predicted attitudes. Vertical collectivism (*β* = 0.20, *p* < 0.001), anthropocentric (*β* = 0.14, *p* < 0.01), environmental apathy (*β* = −34, *p* < 0.001), and self-monitoring (*β* = 0.14, *p* < 0.01) all predicted norms. Horizontal individualism (*β* = 0.17, *p* < 0.001), vertical collectivism (*β* = 0.14, *p* < 0.01), promotion regulatory focus (*β* = −0.11, *p* < 0.05), ecocentric (*β* = 0.30, *p* < 0.001), and environmental apathy (*β* = −22, *p* < 0.001) all significantly predicted perceived behavioral control. Attitudes (*β* = 0.21, *p* < 0.001), norms (*β* = 0.47, *p* < 0.001), and perceived behavioral control (*β* = 0.32, *p* < 0.001) all significantly predicted intention. The full results of the SEM are presented in Table 3 below.

## 5. Discussion

Small individual behaviors can have significant environmental consequences, especially in the areas of energy-saving appliances, solid waste, housing, transportation, water, and food [55]. Thus, it is necessary to understand the complex socio-psychological mechanism behind these “simple” behaviors to benefit public promotion efforts and ultimately contribute to environmental protection. The current study applied the IMBP to investigate the effects of five personality antecedents on young millennials’ and Gen Z’s attitudes, perceived norms, perceived control, and behavioral intention to replace conventional light bulbs with energy-efficient light bulbs. The results showed that environmental value orientation, individualism and collectivism, regulatory focus, and self-monitoring (positively or negatively) predicted attitudes, perceived norms, and perceived control, which subsequently predicted behavioral intention. Even though the descriptive statistics on all IMBP beliefs were relatively favorable, which might not be surprising as young millennials and members of Gen Z are often the driving force in environmental activism and behaviors [11], our results still showed some complex nuances in personality antecedents’ relationships with behavioral intention, even among the “greenest” generations.

The current study made three contributions to the literature and IMBP. First, this study was the first empirical study that explored the predictors of individuals’ intention to switch light bulbs, to the best of our knowledge. While sweeping policy change can make significant impacts on environmental protection, it is also important to examine individual behaviors that can add up quickly. Supporting individual changes and wellbeing “can boost capacity for innovation and collaboration, and ultimately lead to more effective solutions to social and environmental challenges” [56]. Second, previous IMBP research has largely examined health behaviors [13], and the current project extended the application of the model in an environmental-protection context. Moreover, previous studies on environmental protection have examined the associations between a variety of social, behavioral, and psychological factors and environmental protection behaviors, but most of these studies were data-driven rather than theoretically grounded [51]. The theoretical approach contributed to the literature on environmental wellbeing by rendering a more systematic and comprehensive understanding of relevant factors. We further expanded on several findings in our study, and these findings have some practical implications for scholars, policymakers, and environmental activists.

### 5.1. Does CFC Matter?

Contrary to consistent findings in the current literature, CFC did not predict any of the three IMBP beliefs. This could be related to the high CFC values of our participants, namely young millennials and members of Gen Z. Our descriptive findings showed that the average CFC scores were high, with a very small margin of variance. This was consistent with findings from several other studies on millennials and younger adults. For example, Webster and Ma [57] found that younger adults were more likely to be future-oriented than older adults. If millennials have consistently higher consideration of future consequences overall, environmental campaign designers could emphasize the long-term benefits of performing environmentally friendly behaviors when developing messages targeting young millennials and Gen Z. However, it is also possible that CFC was not a personality antecedent of this specific environmentally friendly behavior (i.e., switching to energy-efficient light bulbs), because this action was not perceived as something that could have significant impacts for the future. Whatever the case might be, future research should examine the influences of CFC on specific types of environmentally friendly behaviors, such as adoption versus cessation behaviors or one-time versus continuous behaviors.

### 5.2. Collectivism-Individualism Relationship

The results showed that vertical collectivism was positively related to all three IMBP beliefs, and that horizontal collectivism was positively related to attitudes. These findings aligned with the results from previous studies [35]. We suggest that it is important for media practitioners and environmental activists to incorporate a sense of vertical collectivism when promoting environmental protection behaviors in the future. For one, the messages should not only focus on the community of which the individual is a part, but also the broader “others”. More specifically, to promote with the notion of vertical collectivism, messages that appeal to community hierarchies and focus on authoritative voices on the subject might be more effective in persuading younger adults on environmentally friendly behaviors. It might be more effective to include messages from specific well-respected environmental activists, such as Greta Thunberg, than messages appealing to “you are an equal part of the environment-protection efforts” (i.e., horizontal collectivism). Interestingly, the results also indicated that horizontal individualism was positively correlated with perceived behavioral control, and vertical individualism was not significantly related to any of the three IMBP beliefs, both of which were not consistent with our expectations. Such findings were similar to what Cho et al. [35] found in their study, in which they hypothesized that horizontal and vertical individualism would have indirect negative relationships with environmental commitment, but did not find empirical supports. These findings suggested that people do not necessarily ignore environmental protection and wellbeing simply because they are individualists. Individualism is on the rise globally [58], so it is important for future research to examine how to motivate individualists who value independence, free expression, and looser interpersonal relationships to protect the environment [59]. The effective messages might need to focus on personal achievements and individual status for the ever-growing individualistic adults. Moreover, there are many disagreements surrounding the relationships between individualism and collectivism: some view the two “as opposites of a single continuum, whereas others argue that the two are independent constructs” [60]. We suggest that when it comes to environmental protection, perhaps individualism and collectivism are not the “flip side of a coin”, but rather are two separate factors. The messages targeting cultural orientation should not be opposing, but rather complementary to each other, which could potentially be appealing to both individualists and collectivists. Future studies and efforts should examine effective messages that appeal to community building without compromising the sense of individual achievements.

### 5.3. Apathy, Anthropocentrism, and Ecocentrism

The results revealed some interesting findings on the three dimensions of environmental value orientation. First, environmental apathy was a consistently strong predictor of the IMBP beliefs across the board. This finding suggested that individuals who lack the motivation to protect the environment are not likely to have positive attitudes and other beliefs towards energy-saving behaviors. Even within a population with such an environmentally-friendly mindset, we still see a large number of participants who expressed a high level of environmental apathy. Apathy or indifference towards environmental issues may be caused by inadequate environmental education, lack of funding and ineffective law enforcement for environmental issues, and low priority of environmental conservation by the government [61]. Several articles have called environmental apathy one of the biggest threats to environmental protection efforts [62], and researchers have examined the personality traits associated with environmental apathy [63]. Some effective ways to curb apathy might be education and policy changes [64], but many are still looking for the answer, and we should pay attention to apathy in future research. Second, unlike environmental apathy, ecocentric and anthropocentric values were not decisive predictors of all three IMBP beliefs. Specifically, the anthropocentric value was associated with perceived norms, while the ecocentric value was not. These findings aligned with the previous finding that “rational and self-oriented rather than emotional and others-oriented motives lead millennials to act pro-environmentally actions” [65]. The predictive power of anthropocentric value may depend on the perceived benefits and costs of the given behavior. Anthropocentric individuals believe that nature should be protected because it contributes to human materialistic goals, and they are not likely to act to protect the environment if such actions threaten human-centered values (e.g., quality of life) [44]. This suggested that simply relying on other-oriented arguments might not be sufficient to promote environmentally friendly behaviors, and that combining those arguments with financial or comfort incentives might be most persuasive.

### 5.4. IMBP Beliefs and Intention

The results indicated that perceived norms explained a larger proportion of variances in intention than attitudes and other personality antecedents. This finding was different from many other studies on IMBP, in which attitudes were usually the strongest predictor [15,66]. Such a finding suggested that young millennials and Gen Z place a strong emphasis on “face” when it comes to environmental protection. Just as status motivations were important in luxury green car purchases [36], a recognition of status might be a useful facilitator in promoting environmentally friendly behaviors among young millennials and Gen Z. Moreover, the results showed that self-monitoring was positively related to attitudes and perceived norms, which meant that people who were more concerned about their expressive behaviors’ social and contextual appropriateness were more likely to engage in energy-saving behaviors. Based on these findings, we suggest that environmental campaign designers or media practitioners consider developing a series of social media challenges. In these challenges, a certain social media badge can be given to those who film themselves switching to more energy-efficient light bulbs and host social media competitions for creative content related to the behavioral change. Such actions would help young adults, who are already social media savvy, gain social media approval and appraisal, and such mediated gratifications are highly important for this population [67]. Such social media campaigns may ultimately lead to the perception that switching light bulbs is a socially appropriate and approved behavior and motivate young millennials and members of Gen Z to act and make changes.

### 5.5. Limitations and Directions for Future Studies

First, the current study focused on intention instead of actual behavior as the outcome. According to the IMBP, intention does not always translate into action, and the translation is moderated by individuals’ skills and environmental constraints [66]. For example, young millennials and members of Gen Z may live in dorms, and do not get to choose which light bulbs to use in their rooms. It is also possible that young millennials and members of Gen Z, especially those living in rural areas, do not have access to energy-efficient light bulbs (e.g., some local stores do not carry LEDs). Although the current study results suggest that young millennials and Gen Z have favorable attitudes and strong intentions to replace conventional light bulbs with energy-efficient options, more studies are needed to assess the intention–behavior relationships and the effects of skills and environmental constraints. In other words, future studies should explore what and how environmental constraints affect energy-saving behaviors. Future studies may also apply the IMBP to explore other environmental protection behaviors more systematically and comprehensively. Second, the data collection might have posed some self-selection bias. When we recruited our participants, the large pool of participants had the option to select from a variety of research studies. Our study’s participants could have had more heightened attention towards environmental issues, and our findings should be interpreted within this limitation. Third, we noted several limitations related to the homogenous demographic characteristics of our sample. Specifically, our sample included a significant proportion of participants who might have come from a higher socioeconomic status. We recruited participants from a university in the United States, which had considerably more financial resources to complete the actions (e.g., purchasing energy-saving light bulbs) measured in the survey compared to many people from a lower socioeconomic class. Moreover, our sample had an overproportion of women, who might have reported higher scores on various measures of altruism and agreeableness. These respondents might be more willing to adopt a behavioral change if it is defined as being “for the greater good”. Thus, future research should consider recruiting more generalizable samples and include and measure the influences of relevant demographic factors, such as socioeconomic status. Lastly, there could be potential social desirability bias in our responses, as respondents might appear more environmentally friendly and conscious when it comes to conservation behaviors. These potential social-desirability biases have been documented in previous environmental research [68]. Future research might consider including a measure that assesses the magnitude or certainty of these beliefs.

## 6. Conclusions

Guided by the IMBP, the current study examined the relationships between five personality antecedents and young millennials’ and Gen Z’s attitudes, perceived norms, and control beliefs about replacing conventional light bulbs with energy-efficient options, as well as how these beliefs subsequently predicted the behavioral intention. It is important to understand that there is no “magic bullet” to promote environmentally friendly behaviors, even within a relatively homogenous population. We hope our findings help researchers, policymakers, environmental activists, and business professionals gain a more insightful understanding of this “greenest” generation, and potentially help this generation truly be the force of the changes.

## Figures and Tables

**Table 1:** EFA measurement results.

Latent Variable	Item	Factor Loading	Latent Variable	Item	Factor Loading	Latent Variable	Item	Factor Loading
Intention	INT1	0.79	Vertical Collectivism	VC1	0.58	Ecocentric	VO1	0.57
	INT2	0.89		VC2	0.72		VO2	0.66
	INT3	0.92		VC3	0.63		VO3	0.59
	INT4	0.90		VC4	0.68		VO4	0.50
Attitudes	ATT1	0.40	Consideration of Future Consequences	CFC1	0.42		VO5	0.59
	ATT2	0.82		CFC2	0.44		VO6	0.59
	ATT3	0.72		CFC3	0.68		VO7	0.72
	ATT4	0.84		CFC4	0.60		VO8	0.59
	ATT5	0.85		CFC6	0.40		VO9	0.68
	ATT6	0.67		CFC7	0.41		VO10	0.70
	ATT7	0.82		CFC9	0.63		VO11	0.41
	ATT8	0.63		CFC10	0.68		VO12	0.46
Norms	NORM1	0.82		CFC11	0.62	Anthropocentric	VO13	0.55
	NORM2	0.91		CFC12	0.40		VO15	0.46
	NORM3	0.83	Promotion	RF3	0.61		VO16	0.53
Perceived Control	PBC1	0.70		RF7	0.51		VO17	0.51
	PBC2	0.86		RF9	0.41		VO18	0.54
	PBC3	0.67		RF10	0.73		VO19	0.51
Horizontal Individualism	HI1	0.74		RF11	0.40		VO20	0.44
	HI2	0.60	Prevention	RF2	0.81		VO21	0.47
	HI3	0.60		RF4	0.67		VO23	0.46
	HI4	0.60		RF5	0.40		VO24	0.46
Vertical Individualism	VI1	0.58		RF6	0.75	Environmental Apathy	VO25	0.73
	VI2	0.71		RF8	0.70		VO26	0.63
	VI3	0.63					VO27	0.72
	VI4	0.57					VO28	0.67
Horizontal Collectivism	HC1	0.66					VO29	0.56
	HC2	0.73					VO30	0.73
	HC3	0.50					VO31	0.77
	HC4	0.72					VO32	0.70
							VO33	0.72

Note: All factor loadings were significant at a 0.001 level.

**Table 2:** Measurement covariance results.

	INT	ATT	NOR	PBC	HI	VI	HC	VC	CFC	PRO	PRE	ECC	ACC	EAP	SM
**INT**	−														
**ATT**	0.51 ***	−													
**NOR**	0.65 ***	0.52 ***	−												
**PBC**	0.47 ***	0.41 ***	0.34 ***	−											
**HI**	0.17 ***	0.25 ***	0.17 **	0.29 ***	−										
**VI**	0.01, *p* = 0.86	−0.07, *p* = 07	0.05, *p* = 0.25	0.04, *p* = 0.34	0.30 ***	−									
**HC**	0.15 ***	0.32 ***	0.18 ***	0.21 ***	0.31 ***	−0.03, *p* = 0.63	−								
**VC**	0.14 ***	0.25 ***	0.21 ***	0.17 ***	0.32 ***	0.19 ***	0.46 ***	−							
**CFC**	0.11 ***	0.11 ***	0.09 **	0.09 ***	0.07 *	−0.11 ***	0.13 ***	0.03, *p* = 0.24	−						
**PRO**	−0.07 **	−0.10 ***	−0.05, *p* = 0.06	−0.11 ***	−0.14 ***	−0.03, *p* = 0.10	−0.18 ***	−0.13 ***	−0.06 ***	−					
**PRE**	0.06, *p* = 0.29	0.12 *	0.01, *p* = 0.93	0.02, *p* = 0.73	0.04, *p* = 0.54	−0.19 ***	0.12 *	0.08, *p* = 0.17	0.17 ***	−0.08 *	−				
**ECC**	0.26 ***	0.37 ***	0.19 ***	0.31 ***	0.22 ***	−0.06 *	0.31 ***	0.16 ***	0.14 ***	−0.10 ***	0.05, *p* = 0.24	−			
**ACC**	−0.05, *p* = 0.24	−0.01, *p* = 0.85	0.06, *p* = 0.24	−0.04, *p* = 0.32	0.10 *	0.18 ***	0.05 *p* = 0.15	0.18 ***	−0.14 ***	0.01, *p* = 0.96	−0.13 *	0.02, *p* = 0.50	−		
**EAP**	−0.43 ***	−0.49 ***	−0.31 ***	−0.34 ***	−0.19 **	0.25 ***	−0.30 ***	−0.05, *p* = 30	−0.33 ***	0.13 ***	−0.33 ***	−0.52 ***	0.42 ***	−	
**SM**	0.02, *p* = 0.86	0.20, *p* = 0.11	0.43 ***	−0.02, *p* = 0.83	0.25 *	0.37 ***	−0.07, *p* = 53	0.07, *p* = 0.54	−0.24 **	0.15 *	−0.98 ***	0.122, *p* = 0.20	0.38 ***	0.32 *	−

Notes: *** significant at 0.001 level, ** significant at 0.01 level, * significant at 0.05 level. INT = intention, ATT = attitudes, NOR = norms, PBC = perceived behavioral control, HI = horizontal individualism, VI = vertical individualism, HV = horizontal collectivism, VC = vertical collectivism, CFC = consideration of future consequences; PRO = promotion regulatory factor, PRE = prevention regulatory factor, ECC = ecocentric, ACC = anthropocentric; EAP = environmental apathy, SM = self-monitoring.

**Table 3:** SEM path coefficients.

**Background Variables**								
**Endogenous Variable**	**Exogenous Variable**	**Path Coefficient**	**Endogenous Variable**	**Exogenous Variable**	**Path Coefficient**	**Endogenous Variable**	**Exogenous Variable**	**Path Coefficient**
Attitudes	CFC	−0.06, *p* = 0.18	Norms	CFC	0.03, *p* = 0.46	Perceived Control	CFC	−0.01, *p* = 0.78
	HI	0.09, *p* = 0.06		HI	0.04, *p* = 0.36		HI	0.17 ***
	HC	0.09 *		HC	0.01, *p* = 0.87		HC	−0.04, *p* = 0.44
	VI	−0.08, *p* = 0.07		VI	0.04, *p* = 0.39		VI	0.04, *p* = 0.39
	VC	0.21 ***		VC	0.20 ***		VC	0.14 **
	PROM	−0.03, *p* = 0.57		PROM	−0.02, *p* = 0.75		PROM	−0.11 *
	PREV	0.01, *p* = 0.76		PREV	−0.03, *p* = 0.49		PREV	−0.06, *p* = 0.18
	ECC	0.21 ***		ECC	−0.01. *p* = 0.83		ECC	0.30 ***
	ACC	0.09, *p* = 0.06		ACC	0.14 **		ACC	−0.02, *p* = 0.73
	EAP	−0.38 ***		EAP	−0.34 ***		EAP	−0.22 ***
	SM	0.09 *		SM	0.14 **		SM	−0.04, *p* = 0.39
**IMBP Variables**								
**Endogenous Variable**	**Exogenous Variable**	**Path Coefficient**						
Intention	Attitudes	0.21 ***						
	Norms	0.47 ***						
	Perceived Control	0.32 ***						

Notes: *** significant at 0.001 level, ** significant at 0.01 level, * significant at 0.05 level. HI = horizontal individualism, VI = vertical individualism, HV = horizontal collectivism, VC = vertical collectivism, CFC = consideration of future consequences, PROM = promotion regulatory focus, PREV = prevention regulatory focus, ECC = ecocentric, ACC = anthropocentric; EAP = environmental apathy, SM = self-monitoring.

## Data Availability

The data presented in this study are available on request from the corresponding author. The data are not publicly available due to the ongoing nature of the project.

## Supplementary Materials

The following are available online at https://www.mdpi.com/article/10.3390/ijerph182413104/s1, File S1: Full List of Items in Variables.
